# Supplementary material for: Tailored-Made Polydopamine Nanoparticles to Induce Ferroptosis in Breast Cancer Cells in Combination with Chemotherapy
Source: Int J Mol Sci. 2021 Mar 19;22(6):3161. doi: 10.3390/ijms22063161 (PMC8003616; doi:10.3390/ijms22063161)
Supplement: Supplementary file 1 [file ijms-22-03161-s001.zip › Supplementary material/Supplementary Material.docx]

Tailored-made polydopamine nanoparticles to induce ferroptosis in breast cancer cells in combination with chemotherapy

Celia Nieto, Milena A. Vega and Eva M. Martín del Valle

Supplementary Material

Calculation regarding the amount of Fe^3+^ loaded to PDA NPs as free cation at pH 4.5

Where:

*a =* Free cation Fe^3+^ concentration


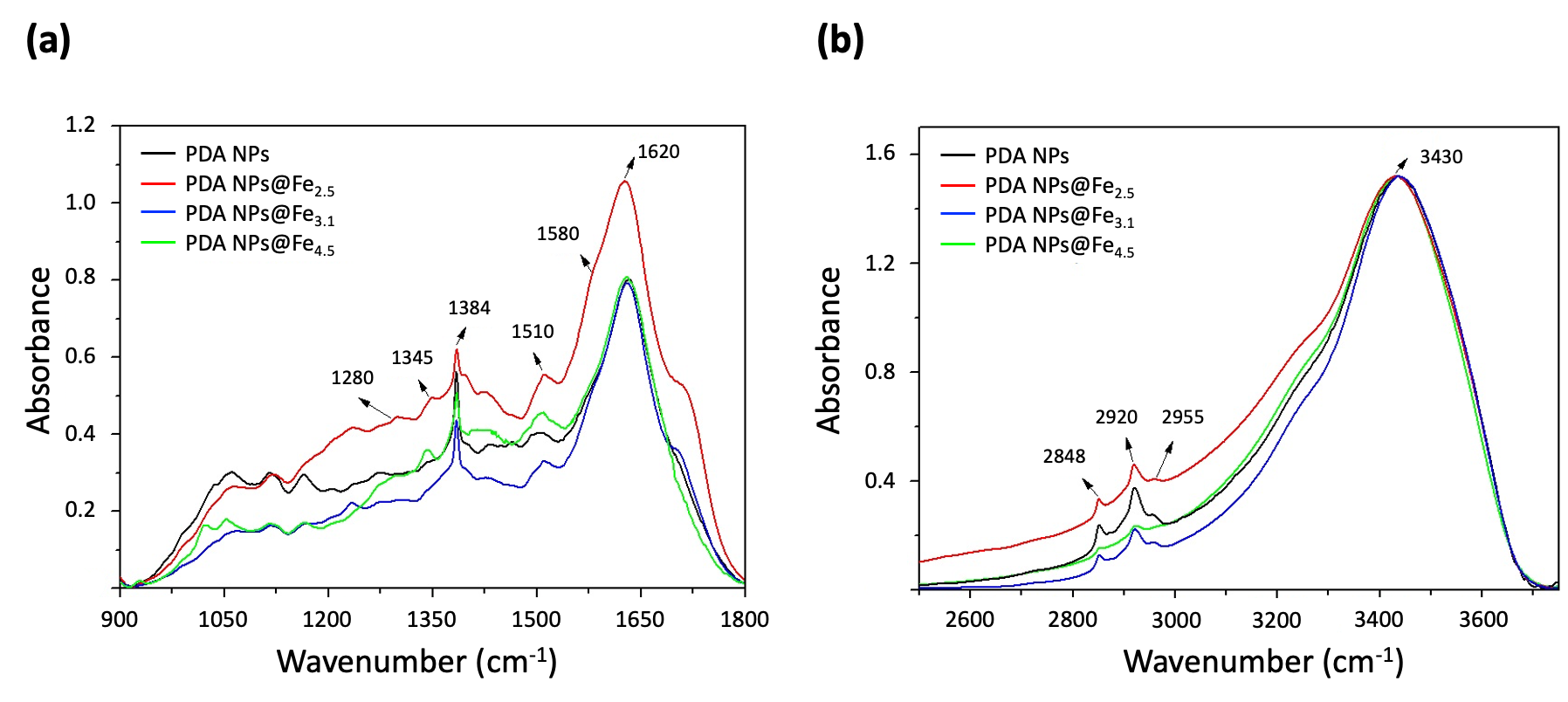


**Figure S1**. IR spectra of PDA NPs and all PDA NPs@Fe in the **(a)** 900-1800 cm^-1^ and **(b)** 2500-3700 cm^-1^ ranges.


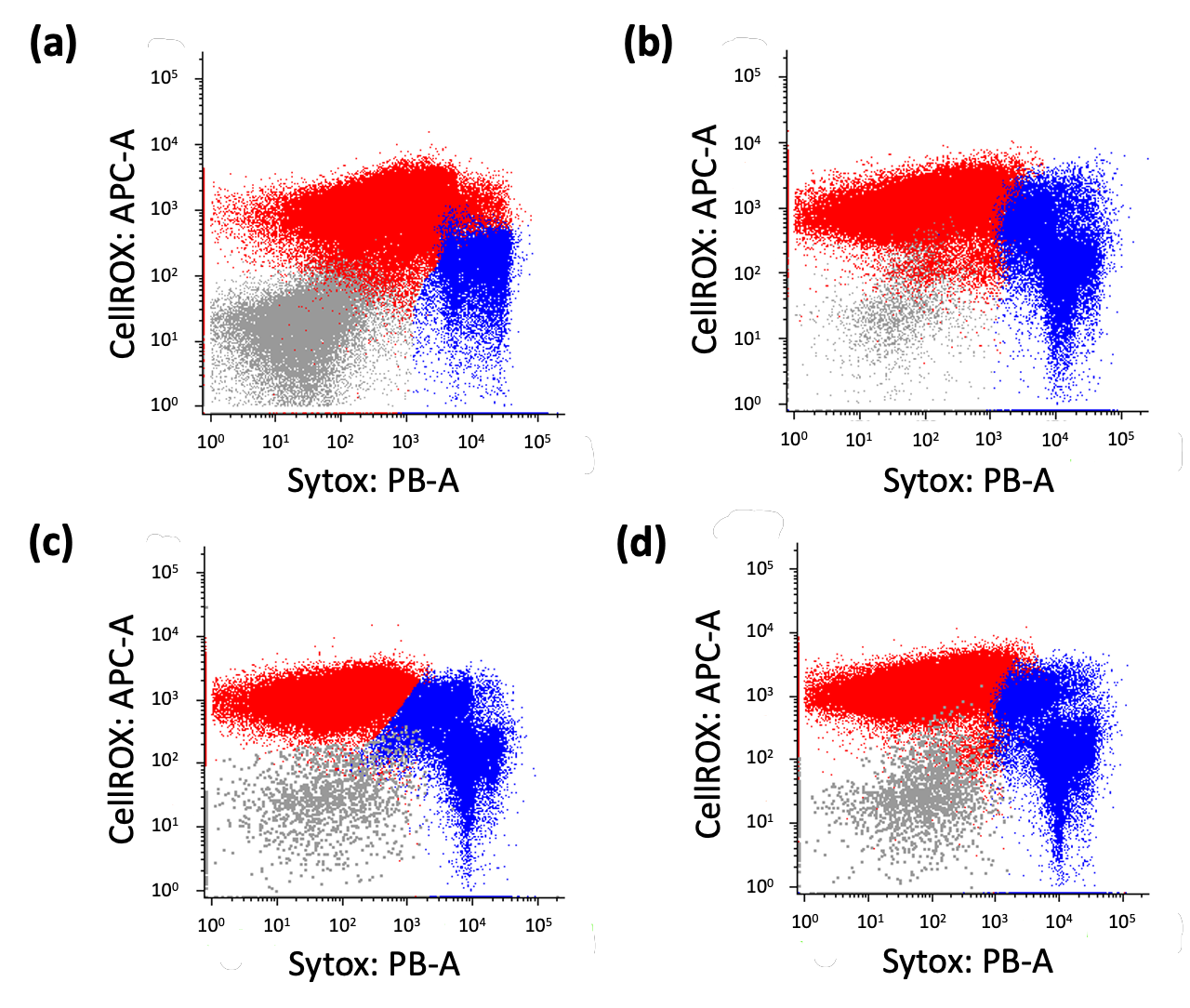


**Figure S2**. Analysis of ROS production *in vitro* through FACS 48 h after BT474 cell treatment with **(a)** DOX (0.3 µM), **(b)** PDA NPs@Fe_2.5_, **(c)** PDA NPs@Fe_3.1_ and **(d)** PDA NPs@Fe_4.5_ (0.035 mg/mL).

**Table S1.** Viability rate values obtained in the MTT assays performed after treating **(a)** BT474 and **(b)** HS5 cells with PDA NPs and the different PDA NPs@Fe (0.035 mg/mL). These values have been the ones represented in Figure 3.

**(a)**

| **Time** | **Control** | **PDA NPs** | **PDA NPs@Fe_2.5_** | **PDA NPs@Fe_3.1_** | **PDA NPs@Fe_4.5_** |
| --- | --- | --- | --- | --- | --- |
| **24 h** | 100 ± 1.1% | 71.6 ± 0.4% | 52.2 ± 1.4% | 52.5 ± 1.9% | 63.6 ± 0.7% |
| **48 h** | 100 ± 1.3% | 67.5 ± 0.6% | 46.6 ± 2.8% | 48.4 ± 1.4% | 58.4 ± 1.4% |
| **72 h** | 100 ± 0.5% | 63.6 ± 1.9% | 43.3 ± 1.4% | 45.6 ± 1.5% | 52.8 ± 1.3% |

**(b)**

| **Time** | **Control** | **PDA NPs** | **PDA NPs@Fe_2.5_** | **PDA NPs@Fe_3.1_** | **PDA NPs@Fe_4.5_** |
| --- | --- | --- | --- | --- | --- |
| **24 h** | 100 ± 1.0% | 91.1 ± 1.2% | 87.8 ± 1.4% | 94.0 ± 0.6% | 85.8 ± 1.8% |
| **48 h** | 100 ± 1.4% | 85.0 ± 2.6% | 85.3 ± 2.8% | 71.8 ± 1.7% | 67.1 ± 1.3% |
| **72 h** | 100 ± 0.8% | 78.1 ± 1.2% | 80.1 ± 1.4% | 64.4 ± 1.3% | 62.5 ± 0.8% |

**Table S2.** Viability rate values obtained in the MTT assays performed after treating **(a-b)** BT474 and **(c-d)** HS5 cells with DOX (0.3-1 µM), PDA NPs@DOX^W^ and PDA NPs@DOX^A^ (0.035 mg/mL). These values have been the ones represented in Figure 4.

**(a)**

| **Time** | **Control** | **DOX (0.3 µM)** | **DOX (0.6 µM)** | **DOX (1 µM)** | **PDA NPs@DOX^W^_0.3_** | **PDA NPs@DOX^W^_0.6_** | **PDA NPs@DOX^W^_1_** |
| --- | --- | --- | --- | --- | --- | --- | --- |
| **24 h** | 100 ± 0.7% | 63.1 ± 0.6% | 53.6 ± 1.3% | 47.5 ± 2.7% | 52.0 ± 2.2% | 43.9 ± 1.9% | 38.3 ± 1.2% |
| **48 h** | 100 ± 0.4% | 25.5 ± 2.3% | 13.2 ± 1.8% | 9.4 ± 2.1% | 44.1 ± 1.8% | 34.3 ± 0.7% | 32.4 ± 1.3% |
| **72 h** | 100 ± 1.6% | 18.4 ± 1.2% | 6.5 ± 1.0% | 4.6 ± 1.4% | 39.7 ± 1.3% | 31.7 ± 1.9% | 26.8 ± 0.7% |

**(b)**

| **Time** | **Control** | **PDA NPs@DOX^A^_0.3_** | **PDA NPs@DOX^A^_0.6_** | **PDA NPs@DOX^A^_1_** |
| --- | --- | --- | --- | --- |
| **24 h** | 100 ± 0.8% | 64.4 ± 1.5% | 57.7 ± 1.4% | 50.1 ± 1.6% |
| **48 h** | 100 ± 1.4% | 60.2 ± 1.2% | 53.2 ± 1.7% | 45.5 ± 2.5% |
| **72 h** | 100 ± 0.9% | 54.8 ± 2.3% | 41.4 ± 1.9% | 36.3 ± 2.0% |

**(c)**

| **Time** | **Control** | **DOX (0.3 µM)** | **DOX (0.6 µM)** | **DOX (1 µM)** | **PDA NPs@DOX^W^_0.3_** | **PDA NPs@DOX^W^_0.6_** | **PDA NPs@DOX^W^_1_** |
| --- | --- | --- | --- | --- | --- | --- | --- |
| **24 h** | 100 ± 2.1% | 73.3 ± 1.3% | 57.8 ± 2.0% | 49.7 ± 1.3% | 90.8 ± 1.7% | 87.7 ± 1.1% | 80.6 ± 0.9% |
| **48 h** | 100 ± 0.8% | 22.7 ± 1.7% | 6.1 ± 1.4% | 1.9 ± 1.5% | 61.6 ± 2.5% | 51.4 ± 1.3% | 44.8 ± 1.5% |
| **72 h** | 100 ± 1.4% | 12.9 ± 0.8% | 2.2 ± 1.5% | 0.2 ± 3.2% | 46.9 ± 2.6% | 38.2 ± 1.7% | 32.5 ± 1.5% |

**(d)**

| **Time** | **Control** | **PDA NPs@DOX^A^_0.3_** | **PDA NPs@DOX^A^_0.6_** | **PDA NPs@DOX^A^_1_** |
| --- | --- | --- | --- | --- |
| **24 h** | 100 ± 1.3% | 88.9 ± 1.0% | 82.2 ± 1.3% | 73.9 ± 0.9% |
| **48 h** | 100 ± 1.9% | 81.4 ± 2.2% | 72.2 ± 2.5% | 68.5 ± 2.0% |
| **72 h** | 100 ± 0.7% | 73.6 ± 1.6% | 64.0 ± 1.7% | 60.8 ± 1.5% |

**Table S3.** Viability rate values obtained in the MTT assays performed after treating **(a-b)** BT474 and **(c-d)** HS5 cells with PDA NPs@Fe/DOX^W^ and PDA NPs@Fe/DOX^A^ (0.035 mg/mL). These values have been the ones represented in Figure 5. Control viability rates were considered 100% in all cases.

**(a)**

| **Time** | **Fe_2.5_ DOX^W^_0.3_** | **Fe_2.5_ DOX^W^_0.6_** | **Fe_2.5_ DOX^W^_1_** | **Fe_3.1_ DOX^W^_0.3_** | **Fe_3.1_ DOX^W^_0.6_** | **Fe_3.1_ DOX^W^_1_** | **Fe_4.5_ DOX^W^_0.3_** | **Fe_4.5_ DOX^W^_0.3_** | **Fe_4.5_ DOX^W^_0.3_** |
| --- | --- | --- | --- | --- | --- | --- | --- | --- | --- |
| **24 h** | 46.4 ± 1.4% | 34.0 ± 2.5% | 32.3 ± 1.6% | 39.5 ± 2.2% | 27.1 ± 3.7% | 25.8 ± 1.9% | 45.2 ± 0.6% | 36.0 ± 0.4% | 31.0 ± 0.2% |
| **48 h** | 37.2 ± 1.4% | 25.8 ± 3.5% | 22.5 ± 2.5% | 31.9 ± 1.1% | 18.1 ± 0.7% | 16.1 ± 1.7% | 37.8 ± 1.0% | 31.7 ± 1.4% | 26.6 ± 0.5% |
| **72 h** | 35.2 ± 2.1% | 21.3 ± 2.1% | 16.1 ± 1.9% | 26.9 ± 0.4% | 9.2 ± 1.4% | 6.7 ± 1.3% | 34.2 ± 1.1% | 29.9 ± 0.5% | 19.4 ± 0.7% |

**(b)**

| **Time** | **Fe_2.5_ DOX^W^_0.3_** | **Fe_2.5_ DOX^W^_0.6_** | **Fe_2.5_ DOX^W^_1_** | **Fe_3.1_ DOX^W^_0.3_** | **Fe_3.1_ DOX^W^_0.6_** | **Fe_3.1_ DOX^W^_1_** | **Fe_4.5_ DOX^W^_0.3_** | **Fe_4.5_ DOX^W^_0.3_** | **Fe_4.5_ DOX^W^_0.3_** |
| --- | --- | --- | --- | --- | --- | --- | --- | --- | --- |
| **24 h** | 51.8 ± 1.4% | 40.2 ± 1.4% | 38.4 ± 1.2% | 47.2 ± 2.3% | 38.6 ± 1.7% | 34.7 ± 2.2% | 54.5 ± 1.3% | 42.0 ± 2.1% | 39.2 ± 2.0% |
| **48 h** | 44.2 ± 1.2% | 35.7 ± 2.4% | 28.6 ± 1.4% | 40.8 ± 1.0% | 31.6 ± 0.9% | 23.8 ± 1.5% | 46.5 ± 1.7% | 40.9 ± 1.9% | 33.2 ± 2.5% |
| **72 h** | 42.7 ± 1.9% | 29.0 ± 2.1% | 24.8 ± 1.5% | 37.9 ± 2.2% | 24.9 ± 1.5% | 20.2 ± 1.3% | 40.8 ± 1.9% | 35.4 ± 1.7% | 27.2 ± 1.7% |

**(c)**

| **Time** | **Fe_2.5_ DOX^W^_0.3_** | **Fe_2.5_ DOX^W^_0.6_** | **Fe_2.5_ DOX^W^_1_** | **Fe_3.1_ DOX^W^_0.3_** | **Fe_3.1_ DOX^W^_0.6_** | **Fe_3.1_ DOX^W^_1_** | **Fe_4.5_ DOX^W^_0.3_** | **Fe_4.5_ DOX^W^_0.3_** | **Fe_4.5_ DOX^W^_0.3_** |
| --- | --- | --- | --- | --- | --- | --- | --- | --- | --- |
| **24 h** | 82.4 ± 1.4% | 75.3 ± 2.5% | 67.8 ± 1.6% | 84.9 ± 1.4% | 75.4 ± 1.8% | 73.5 ± 1.4% | 80.2 ± 1.7% | 72.2 ± 1.4% | 65.3 ± 0.7% |
| **48 h** | 71.3 ± 1.4% | 62.5 ± 3.5% | 56.8 ± 2.5% | 60.7 ± 1.6% | 49.3 ± 1.7% | 45.7 ± 1.6% | 56.3 ± 1.2% | 45.6 ± 1.4% | 42.9 ± 0.9% |
| **72 h** | 65.0 ± 2.1% | 48.8 ± 2.1% | 45.9 ± 1.9% | 50.5 ± 1.5% | 35.7 ± 2.1% | 27.4 ± 2.1% | 52.9 ± 1.6% | 44.1 ± 1.7% | 35.1 ± 0.8% |

**(d)**

| **Time** | **Fe_2.5_ DOX^W^_0.3_** | **Fe_2.5_ DOX^W^_0.6_** | **Fe_2.5_ DOX^W^_1_** | **Fe_3.1_ DOX^W^_0.3_** | **Fe_3.1_ DOX^W^_0.6_** | **Fe_3.1_ DOX^W^_1_** | **Fe_4.5_ DOX^W^_0.3_** | **Fe_4.5_ DOX^W^_0.3_** | **Fe_4.5_ DOX^W^_0.3_** |
| --- | --- | --- | --- | --- | --- | --- | --- | --- | --- |
| **24 h** | 84.1 ± 1.5% | 77.4 ± 2.0% | 70.5 ± 1.6% | 83.7 ± 1.5% | 79.9 ± 0.3% | 73.4 ± 0.3% | 89.4 ± 0.9% | 79.0 ± 1.7% | 74.5 ± 1.8% |
| **48 h** | 76.6 ± 1.8% | 67.9 ± 1.7% | 61.9 ± 1.6% | 65.8 ± 1.4% | 63.9 ± 1.5% | 58.4 ± 1.5% | 72.3 ± 3.2% | 60.0 ± 1.5% | 51.5 ± 0.7% |
| **72 h** | 69.2 ± 2.6% | 59.4 ± 2.7% | 52.9 ± 1.9% | 62.1 ± 1.7% | 57.5 ± 1.3% | 45.4 ± 1.7% | 56.9 ± 1.5% | 47.8 ± 2.7% | 40.1 ± 1.4% |
